# Intersectional Barriers to and Facilitators of Educational Inclusion for Persons With Disabilities in Low‐ and Middle‐Income Countries: A Systematic Literature Review

**DOI:** 10.1002/hsr2.73349

**Published:** 2026-10-14

**Authors:** Yusufu Simra, Shani Omari, Hussein Mwanjali

**Affiliations:** ^1^ Department of Public Health St. Francis University College of Health and Allied Sciences (SFUCHAS) Ifakara Morogoro Tanzania; ^2^ Department of Research Policy and Innovation, Tanzania Inclusion and Disability Foundation Dar es Salaam Tanzania

**Keywords:** educational inclusion, facilitators, intersectional barriers, low‐ and middle‐income countries, persons with disabilities, systematic literature review

## Abstract

**Background:**

in low‐ and middle‐income countries, the exclusion of persons with disabilities from education is influenced by a combination of structural, environmental, and psychosocial constraints. These challenges are further compounded by intersecting factors such as gender, socioeconomic status, and geographic location, which deepen existing inequalities. Gaining insight into these overlapping dynamics is essential for advancing inclusive education.

**Methods:**

A systematic literature search was conducted across Google Scholar, PubMed/MEDLINE, ERIC, and African Journals Online, yielding 132 records. This review was retrospectively registered with PROSPERO (registration number CRD420261360472). After screening titles and abstracts, assessing full text eligibility, and conducting quality appraisal using the Mixed Methods Appraisal Tool (MMAT), 23 studies were included in the review. The data were then analyzed and synthesized using a narrative thematic approach.

**Results:**

The findings highlight a consistent gap between policy and implementation, alongside insufficient infrastructure and pervasive stigma that hinder inclusive education. Intersectional factors were found to amplify disadvantage, particularly among girls and individuals in rural settings. At the same time, community‐based interventions and the use of assistive technologies were identified as important enablers of inclusion.

**Conclusion:**

The study demonstrates that educational exclusion arises from interconnected structural and societal influences rather than disability in isolation. Effectively addressing these challenges requires tackling intersectional barriers through systemic reforms, inclusive teaching practices, and active community involvement. Achieving sustainable inclusion will depend on a dual strategy that addresses both individual needs and broader systemic issues.

## 1 Introduction

The exclusion of individuals with disabilities from education in low‐ and middle‐income countries (LMICs) remains a critical human rights and development concern in the 21st century. Although education is recognized as a fundamental right under Article 26 of the Universal Declaration of Human Rights and Article 24 of the United Nations Convention on the Rights of Persons with Disabilities (UNCRPD), many individuals with disabilities continue to face significant marginalization [1, 2, 3]. Current global estimates indicate that between 180 and 240 million children live with disabilities, with approximately 90% residing in LMICs [4, 5]. In several African countries, school attendance among children with disabilities is alarmingly low often below 10% and these children are far more likely than their non‐disabled peers to have never accessed formal education [3, 6, 7]. This widespread exclusion is not only a social injustice but also a key driver of persistent poverty, as limited access to education restricts employment opportunities and undermines economic independence [6, 8].

Research consistently shows that educational inclusion is shaped by a complex interplay of systemic, environmental, and psychosocial factors [2, 9]. At the systemic level, a notable gap often exists between inclusive education policies and their practical implementation [1, 9, 10]. While many LMICs have adopted inclusive frameworks, these are frequently undermined by insufficient funding, limited infrastructure, and a shortage of trained personnel. Environmental barriers further compound the problem, as many schools lack essential accessibility features such as ramps, adapted sanitation facilities, and inclusive transportation systems. As a result, children with physical or sensory impairments are often unable to participate fully in schooling [5, 11, 12]. Even when enrolled, they commonly encounter inadequate instructional support and curricula that do not accommodate diverse learning needs [1, 11, 13].

Recent scholars increasingly emphasize the intersectional dimensions of educational exclusion. Disability intersects with other social determinants including gender, socioeconomic status, and geographic location to produce layered forms of disadvantage [7, 9, 14]. For example, in sub‐Saharan Africa, girls with disabilities frequently experience compounded discrimination, as cultural expectations may prioritize domestic responsibilities over schooling. Similarly, children in rural areas often remain unidentified or unsupported due to limited access to diagnostic and educational services outside urban centers [14, 15, 16]. Addressing these intersecting influences is essential for achieving Sustainable Development Goal 4 (SDG 4), which calls for inclusive and equitable quality education for all [7, 10, 17].

Over time, perspectives on disability and education in LMICs have shifted from a predominantly medical model where disability is viewed as an individual deficit to a social model that emphasizes the removal of structural and attitudinal barriers [1, 17, 18]. The application of frameworks such as Bronfenbrenner's Ecological Systems Theory has enabled researchers to examine these barriers across multiple levels, including the microsystem (e.g., peer interactions and teacher attitudes), mesosystem (e.g., relationships between families and schools), and macrosystem (e.g., national policies and cultural norms) [9, 11]. Among these, stigma and negative societal perceptions remain particularly entrenched. In many LMIC contexts, disability is still associated with spiritual or cultural interpretations such as curses or punishment, which reinforces exclusionary attitudes [5, 6]. These beliefs often translate into low expectations for learners with disabilities and contribute to social isolation and discrimination, even within higher education environments [11, 19]. It is important to situate these findings within the broader international discourse on inclusive education. Scholars have long cautioned that mere placement in a mainstream classroom does not automatically constitute inclusion as defined under Article 24 of the UNCRPD; genuine inclusion additionally requires meaningful participation, belonging, and pedagogical adaptation [20, 21]. Comparative reviews of global inclusive education research have similarly identified persistent conceptual ambiguity regarding what “inclusion” means across education systems, with definitions ranging from narrow physical placement to broader, rights‐based understandings of full participation [20, 22]. These conceptual tensions are echoed in decolonial critiques, which argue that inclusive education frameworks developed in the Global North are frequently transplanted into LMIC and postcolonial contexts without adequate adaptation to local knowledge systems, resource realities, or cultural values [23, 24]. The present review builds on, and extends, this literature by empirically mapping how such conceptual and structural tensions manifest specifically at the intersection of disability, gender, poverty, and geography in LMIC settings. This review is guided by the central question, how do intersectional factors specifically gender, socioeconomic status, and geographic location shape the environmental, systemic, and psychosocial barriers and facilitators affecting the educational inclusion of persons with disabilities in LMICs? By moving beyond a simplistic understanding of disability, this inquiry seeks to explore how overlapping social identities generate distinct forms of disadvantage and how these can be addressed through targeted interventions [4, 18].

Accordingly, this systematic review has two primary objectives. First, it aims to identify and categorize the physical, systemic, and psychosocial barriers and facilitators influencing educational inclusion across different LMIC contexts, drawing on perspectives from students, parents, and educators [11, 19, 25, 26]. Second, it seeks to examine how factors such as gender and poverty interact to intensify exclusion, thereby offering a clearer understanding of intersectional disadvantage than is typically provided in broader disability research [4, 14, 18].

## 2 Methods

### 2.1 Study Design and Protocol Registration

This study adopted the Preferred Reporting Items for Systematic Reviews and Meta‐Analyses (PRISMA) 2020 guidelines to ensure a transparent, systematic, and replicable process in identifying, screening, and selecting relevant literature on disability, education, exclusion, inclusion, barriers, and facilitators.

This review was initially undertaken as an academic exercise without an a priori intention of external publication; consequently, the review protocol was developed and initial database searches were conducted before formal trial registration was sought. Once the decision was made to submit this work for peer review, the completed protocol was registered with the International Prospective Register of Systematic Reviews (PROSPERO; registration number CRD420261360472). No changes were made to the review question, eligibility criteria, search strategy, or planned synthesis method between protocol development and PROSPERO registration. Because registration occurred retrospectively rather than prospectively, this is acknowledged as a limitation of the review (see Section 6).

### 2.2 Search Strategy

A systematic literature search was conducted across four databases: Google Scholar, PubMed/MEDLINE, ERIC (Education Resources Information Center), and African Journals Online (AJOL). ERIC was included given the education focus of the review question; AJOL was included to improve retrieval of literature from sub‐Saharan African contexts. Searches were conducted between January and April 2025.

The following Boolean search string was applied, with minor syntactic adaptation for each platform:

*(“disability” OR “persons with disabilities” OR “children with disabilities”) AND (“education” OR “schooling” OR “inclusive education”) AND (“barriers” OR “facilitators” OR “exclusion” OR “inclusion”) AND (“low‐income countries” OR “middle‐income countries” OR “LMICs” OR “sub‐Saharan Africa” OR “South Asia” OR “developing countries”)*

Truncation and phrase operators were used where supported by the database interface. Searches were restricted to publications in the English language between January 2018 and December 2025. The reference lists of all included studies were screened for additional eligible sources (backward citation searching). The search strategy did not employ grey literature databases, trial registries, or controlled vocabulary indexing such as MeSH or EMTREE terms. This limits reproducibility and may have resulted in relevant studies being missed, and is reported transparently as a limitation in Section 6.

### 2.3 Identification of Studies and Screening Process

The initial search yielded a total of 132 records. These were compiled for screening. Title and abstract screening were conducted by the lead reviewer against the inclusion and exclusion criteria described in Section 2.4. Given resource constraints, dual independent screening was not undertaken at this stage; this is acknowledged as a limitation. Full text eligibility assessment was also completed by the lead reviewer using a standardized checklist. Where eligibility was uncertain at full‐text stage, the study was retained and a second team member was consulted before a final decision was reached.

The screening process involved two stages. All 132 studies were reviewed based on their titles and abstracts; studies that clearly did not align with the research focus were excluded. After the initial screening, 60 studies were retained for further assessment. Applying the full inclusion and exclusion criteria resulted in the removal of 35 studies, leaving 25 studies for eligibility assessment. This screening and selection process is summarized in the PRISMA 2020 flow diagram (Figure 1).

**Figure 1:**
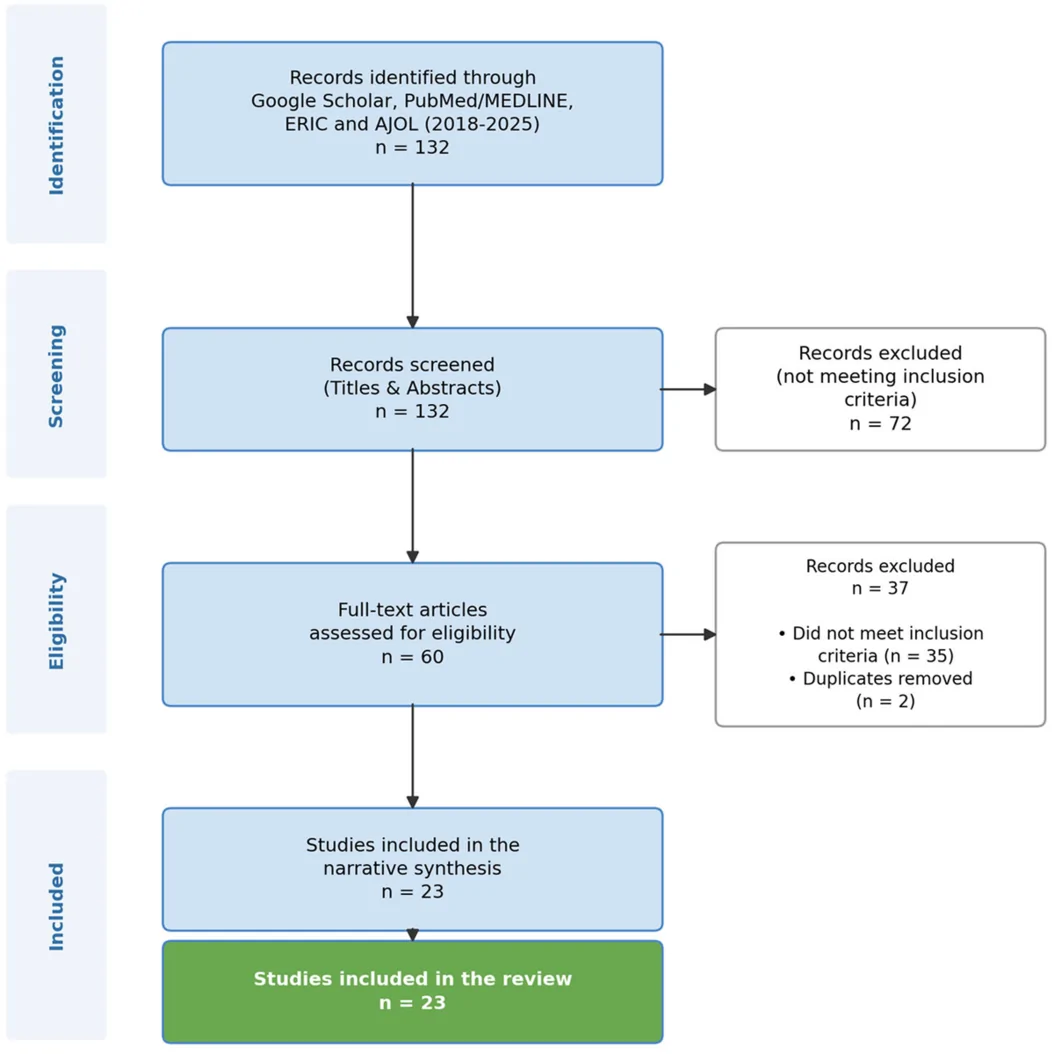
PRISMA 2020 flow diagram illustrating the study selection process. A total of 132 records were identified through database searching. All 132 records were screened by title and abstract, of which 72 were excluded as irrelevant. Sixty records were assessed for full text eligibility. Thirty‐seven were excluded: 35 did not meet inclusion criteria and 2 were identified as duplicates at full text stage. A final total of 23 studies were included in the narrative synthesis.

### 2.4 Inclusion and Exclusion Criteria

Studies were included if they met all of the following criteria: published in the English language; peer‐reviewed journal articles or high‐quality academic theses; directly addressed issues related to disability, inclusive education, or educational inclusion and exclusion in a LMIC context; and published between January 2018 and December 2025.

Studies were excluded if they met any of the following conditions: published before 2018; non‐peer‐reviewed sources such as opinion pieces, book reviews, or non‐academic articles, publications in languages other than English, or studies conducted exclusively in high income country contexts with no comparative LMIC data.

With respect to study design, this review included peer‐reviewed qualitative studies, quantitative cross‐sectional and descriptive studies, mixed methods studies, and published systematic or scoping reviews. Given the nature of the research question which focuses on lived experiences, structural barriers, and contextual facilitators rather than intervention efficacy experimental designs were not expected to constitute a significant portion of the available evidence and none were identified during screening. Grey literature, unpublished theses, conference abstracts, and non‐peer‐reviewed reports were excluded.

### 2.5 Eligibility Assessment

The remaining 25 studies underwent full‐text review to determine their suitability for final inclusion. During this phase, 2 duplicate studies were identified and removed. This resulted in a final sample of 23 studies deemed eligible for inclusion in the systematic review.

### 2.6 Quality Appraisal

The methodological quality of all 23 included studies was assessed using the Mixed Methods Appraisal Tool (MMAT), version 2018 [Hong Q.N. et al., 2018, doi: 10.1177/1558689818795743]. The MMAT was selected for its validated applicability across qualitative, quantitative, and mixed‐methods study designs, reflecting the heterogeneous evidence base of this review.

Each study was assessed against five criteria appropriate to its design type. Consistent with the classification scheme applied in Appendix A, studies were rated as low quality (1–2 criteria met), moderate quality (3–4 criteria met), or high quality (5 criteria met). Of the 23 included studies, 7 were rated high quality, 12 moderate quality, and 4 low quality. Low‐quality studies were retained in the synthesis but their findings were interpreted with caution; none were used as the sole basis for any conclusion drawn in this review. Quality ratings for each of the 23 included studies are reported in full in Appendix A. Background and conceptual references cited elsewhere in this manuscript were not part of the systematically searched and screened evidence base and were therefore not subject to MMAT appraisal. Inter‐rater reliability was not formally assessed, as this review was conducted primarily by a single reviewer, which is reported as a limitation.

### 2.7 Data Extraction and Synthesis

Data from the selected studies were systematically extracted using a standardized extraction template capturing: study objectives, methodology, population and context, key findings related to disability and educational inclusion and exclusion, and barriers and facilitators for disability education in LMICs. A narrative synthesis approach was employed to analyze and integrate findings across the selected studies, identifying common themes, patterns, and gaps in the literature. Narrative synthesis was chosen over meta‐analysis given the methodological heterogeneity of the included studies and the qualitative nature of a substantial portion of the evidence base.

As this review employed narrative (not statistical) synthesis of qualitative and mixed‐methods evidence, no inferential statistical hypothesis testing (e.g., significance testing or *p* values) was performed and no dedicated statistical software package was required; quality‐appraisal scores (MMAT) and study counts are reported descriptively as frequencies and percentages, consistent with SAMPL guidance for reporting non‐inferential summary statistics.

## 3 Results

### 3.1 Characteristics of Included Studies

The 23 included studies span a range of methodological approaches and geographic contexts, collectively addressing intersectional barriers and facilitators influencing the educational inclusion of persons with disabilities (PWDs) within Low‐ and Middle‐Income Countries (LMICs) [4, 9, 14]. This field examines the complex interplay between physical, systemic, and psychosocial factors, with particular emphasis on how disability status intersects with other social determinants such as gender, socioeconomic status (poverty), and geographic location (rural vs. urban settings) [4, 14]. The scope includes various levels of education, from pre‐primary and early childhood development (ECD) to primary, secondary, and higher education [5, 11, 19].

### 3.2 Data Extraction Sources

Data were extracted from a total of 23 sources encompassing various research methodologies. These include systematic and scoping reviews providing evidence on barriers and facilitators in low‐ and middle‐income countries [15]; primary qualitative research capturing the lived experiences of students, parents, and teachers [2, 5, 6, 26, 27]; quantitative and mixed‐methods studies offering statistical insights into enrollment trends, psychosocial impacts, and gendered disparities [10, 12, 14, 26, 27]; and peer‐reviewed studies examining policy frameworks, decolonial perspectives, and higher education inclusion contexts [4, 17].

The literature provides broad geographic coverage across diverse LMIC regions, with a strong focus on Sub‐Saharan Africa and South/Central Asia. Countries represented include Tanzania [4, 5, 19, 26, 27], South Africa [3, 15, 28], Kenya [8, 15], Zimbabwe [6, 15], Malawi [15], Uganda [8, 25], Zambia [8, 15], Ethiopia [2, 14, 16], Nigeria [10], Ghana [2, 16], India [17].

Table 1 summarises the key characteristics of the 23 included studies—country context, disability type, primary barriers, and policy responses. The intersectional dimensions of gender, poverty, and geography that emerge from these data are analysed in detail in Section 3.5.

**Table 1:** Characteristics of the 23 included studies, showing region, country, disability type, intersectional factors, primary barriers, policy responses, and socio‐economic impact.

Region	Country	Disability Type	Intersectional Factors	Primary Barriers	Policy Response	Socio‐Economic Impact
Sub‐Saharan Africa	South Africa	Profound Intellectual Disability/Cerebral Palsy	Age (Adolescents) vs. Economic status	Insufficient teacher training, high transport costs, lack of adapted programs, exclusion from extramural activities.	Education White Paper 6; Teacher Education for Disability Inclusion (TEDI) project; National Strategy for Inclusive Education.	Lifelong dependency, reduced physical development, exclusion from workforce.
Sub‐Saharan Africa	Zimbabwe	General Physical, Sensory, and Intellectual	Gender (Female caregivers) vs. Poverty	High school fees, lack of assistive devices, rural location, cultural norms viewing disability as a curse.	Constitution Amendment No. 20; National Disability Policy (2021).	High dropout rates, permanent exclusion from labor market.
Sub‐Saharan Africa	Tanzania	Hearing, Visual, Physical, and Albinism	Poverty vs. Education level	Low teacher expectations, lack of Braille/sign language skills, superstitious beliefs regarding albinism.	Education and Training Policy (2014); Persons with Disabilities Act (2010).	Poor academic performance, vulnerability to ritual violence.
South Asia	India	Developmental, Learning, Intellectual, Physical	Gender (Girls) vs. Caste/Income	Community stigma against educating girls, lack of remedial services, inaccessible toilets (41%).	Sarva Shiksha Abhiyan; Rights of Persons with Disabilities (RPWD) Act 2016; NEP 2020.	Disabled girls face the highest exclusion, trapped in cycle of poverty.
Sub‐Saharan Africa	Ethiopia & Ghana	Visual, Hearing, Physical	Rural vs. Urban location and Poverty	Psychosocial stigma (bullying), physical barriers (lack of ramps), lack of electricity for remote learning.	Inclusive Education Policy (Ghana); Education Sector Development Program VI (Ethiopia).	Rural‐to‐urban migration for schooling, high dropout rates.
Sub‐Saharan Africa	Uganda	Neurodisabilities (Physical and Intellectual)	Poverty vs. Gender (Clans/Tribes)	Large class sizes, built environment barriers, exclusion from paternal inheritance due to cultural norms.	Community Based Rehabilitation (CBR); teacher training on Ubuntu bulalamu (peer‐to‐peer support).	Lifelong economic marginalization and social isolation.
Sub‐Saharan Africa	Nigeria	Hearing, Visual, Physical	Gender vs. Regional disparities (North vs. South)	Inadequate supportive facilities, lack of teacher training, low utilization of assistive technologies.	National Policy on Special Needs Education 2015; UN CRPD (2010).	High out‐of‐school rates (5‐7 million children), double discrimination for girls.
Caribbean	Dominican Republic	Autism, Cerebral Palsy, Cognitive	Disability vs. Poverty	Social stigma based on charity models, lack of universal accessibility in transport, inconsistent enforcement of labor quotas.	Organic Law No. 5‐13 on Equality of Rights; Interuniversity Inclusion Network.	Significant disparity in higher education attainment (16% vs. 22%).
Sub‐Saharan Africa	Kenya	Complex Communication Needs (CCN)	Rural location vs. Disability	Lack of transport, facilities, and staffing; fear and stigma associated with disability.	Home‐based interventions using Augmentative and Alternative Communication (AAC).	Developmental delays due to lack of engagement outside family unit.
Sub‐Saharan Africa	Zambia	Physical and Cognitive	Poverty vs. Social factors	Financial burden of school fees, lack of equipment, refusal of schools to admit children with disabilities.	Community‐based rehabilitation (CBR) services.	Total exclusion from labor market.
Sub‐Saharan Africa	Malawi	Intellectual Disability	Poverty vs. Access to health services	Social barriers, lack of support from medical professionals, environment not adapted to child's needs.	Not documented in source.	Isolation and limited potential to contribute to community.

Educational exclusion cannot be attributed to disability in isolation; rather, it is shaped by the interaction of multiple, overlapping social identities. Evidence from various regions highlights a compounded disadvantage experienced by girls, where the intersection of gender and disability gives rise to distinct challenges [4]. These include expectations around domestic labor that often take precedence over schooling, as well as limited access to distance learning opportunities during periods of crisis. Geographic location further constrains inclusion, both through the physical distance to educational facilities and a “detection gap” [2, 13, 16], whereby specialized diagnostic and support services are largely concentrated in urban areas. In addition, poverty consistently intensifies the impact of disability. In contexts where social protection systems are weak or absent, families are required to cover the costs of assistive devices and related support, which can lead to complete exclusion from education when financial resources are inadequate [4, 6, 7]. The specific characteristics of these included studies, including their research designs and primary data extraction results, are synthesized and presented in Table 1.

### 3.3 Systemic and Environmental Barriers

Systemic barriers are characterized by a profound policy‐practice gap. While many LMICs have ratified international mandates including the UNCRPD, local implementation is stifled by a lack of decentralized monitoring and insufficient budget allocations [12, 26]. In rural and post‐colonial contexts, the physical environment remains a built barrier, where even basic sanitation and mobility needs are unmet, effectively excluding those with high support needs [7, 12]. Curricula across many of the reviewed contexts were also described as “non‐inclusive” in that they remain rigidly standardized, examination‐driven, and delivered at a single pace and in a single language, leaving little room for differentiated instruction, alternative formats (e.g., Braille, sign language, or simplified text), or assistive‐technology integration [2, 11, 13]. In practice, learners with sensory, intellectual, or communication impairments are expected to meet the same benchmarks as their non‐disabled peers without corresponding adaptations to content, pacing, or assessment method—a mismatch that several of the included studies identify as a key driver of high dropout and low completion rates despite nominal enrollment [2, 13]. The systemic and environmental barriers identified across the included studies, along with their impact on student participation, are synthesized in Table 2.

**Table 2:** Analysis of systemic and environmental barriers to inclusive education in LMIC contexts.

Authors & year	Main aim	Main results obtained
Lawal et al. (2025); Zickafoose et al. (2024)	To evaluate policy implementation and funding barriers in Nigeria and Sub‐Saharan Africa (SSA).	Found that implementation remains highly inadequate due to resource misallocation, lack of assistive technologies, and fragmented data systems.
Abebe et al. (2023)	To explore physical and infrastructural barriers in rural India, Ethiopia, and Ghana.	High enrollment does not equal inclusion; only 41% of schools had accessible toilets, and 37% had ramps. Students in Africa cited non‐inclusive curricula as a major barrier.
Temba et al. (2024)	To examine accessibility in Tanzanian private primary schools.	Found extremely low enrollment (1.03%) due to school owners' reluctance and high operational costs, though retention was high once enrolled.

### 3.4 Psychosocial and Attitudinal Barriers

The literature suggests that, in many contexts, stigma and limited teacher self‐efficacy present more significant barriers to inclusion than physical constraints. The continued dominance of the medical model reinforces this challenge, as it frames disability as an individual deficit requiring correction rather than addressing the shortcomings of the surrounding environment [5, 17]. This perspective often leads to forms of internalized exclusion, where students experience social isolation despite being physically present in the classroom [19]. Evidence from higher education indicates that these patterns extend beyond primary schooling, with students with disabilities frequently reporting exclusion from peer social networks and a persistent lack of adequate academic support systems [11]. The core psychosocial and attitudinal hurdles identified in the literature, alongside their impact on educational equity, are synthesized in Table 3.

**Table 3:** Overview of psychosocial and attitudinal barriers influencing educational exclusion.

Author(s) & Year	Main Aim	Main Results Obtained
Mbatt & Philip (2024); Makwelo et al. (2025)	To assess the psychosocial impact of inclusion on students and teachers.	Teachers often hold low expectations for disabled students, believing they are incapable of academic success, which reduces their commitment to support.
Cosmas (2025); Bani Odeh & Lach (2024)	To explore teachers' conceptualizations of disability and worldwide barrier trends.	Disability is frequently viewed through a tragedy or spiritual lens (e.g., a curse), leading to stigma and peer bullying.
Masuku & Marumolo (2024)	To explore perceptions of inclusive education for children with complex communication needs (CCN).	Parents often mistrust inclusive settings due to fears of their children being bullied or neglected by untrained staff, leading to a preference for segregated special education.

### 3.5 Intersectional Factors: Gender, Poverty, and Geography

Intersectionality plays a critical role in shaping patterns of exclusion. In particular, girls especially in Sub‐Saharan Africa often experience compounded discrimination, where the combined effects of gender and disability push them out of school and into caregiving responsibilities [7, 14]. At the same time, disparities between rural and urban settings further deepen inequality. Learners in remote areas typically have little to no access to specialized services, while those in urban centers may benefit from limited, often fragmented, forms of support [2, 12]. These factors do not operate independently but interact cumulatively. A girl with a disability living in a low‐income rural household, for example, simultaneously faces gendered expectations to prioritise domestic labor over schooling, financial barriers to assistive devices and transport, and geographic isolation from specialized diagnostic or support services such that her likelihood of exclusion is substantially higher than would be predicted by any single factor considered alone [7, 14, 17]. This compounding pattern helps explain why interventions that target only one dimension of disadvantage (for instance, a disability‐specific policy introduced without complementary attention to gender norms, poverty alleviation, or rural service provision) are unlikely, on their own, to achieve meaningful inclusion. The intersectional patterns identified across the included studies are summarized in Table 4.

**Table 4:** Intersectional impact of gender, poverty, and geography on educational access.

Author(s) & Year	Main Aim	Main Results Obtained
Jones et al. (2021)	To explore the intersection of gender, disability, and poverty during COVID‐19.	In Bangladesh and Ethiopia, girls with disabilities were the most disadvantaged, facing higher domestic labor burdens and less access to distance learning.
Hussein (2025)	To analyze the double disadvantage of gender and disability.	Intersectionality creates unique barriers where women with disabilities face heightened marginalization in both education and future employment.
Gull et al. (2025)	To review challenges in higher education for students with disabilities.	Students with disabilities in universities face social isolation and rejection from peer friendship circles, compounded by a lack of academic support.

### 3.6 Facilitators and Interventions

Facilitating inclusion in many LMIC contexts is largely rooted in community‐based approaches. Although advanced assistive technologies offer significant potential, effective practice often depends on the reinforcement of indigenous principles such as Ubuntu, which prioritize collective responsibility and mutual support [6, 16]. However, the existing body of research tends to focus more on adapting the individual learner than on addressing structural constraints. As a result, comparatively less attention has been given to systemic approaches, such as Universal Design for Learning (UDL), that aim to create more inclusive educational environments for all students [3, 8]. The primary facilitators and successful educational interventions discovered in this review are summarized in Table 5.

**Table 5:** Identified facilitators and educational interventions for enhancing inclusion.

Author(s) & Year	Main aim	Main results obtained
Hunt et al. (2025)	To review the effectiveness of educational interventions in LMICs.	Individual level interventions (literacy, numeracy, and handwriting) show positive effects, but there is a critical lack of research on system level changes.
Tafirenyika et al. (2023)	To explore community‐based learning in rural Zimbabwe.	Caregivers are willing to offer free labor, food, and local materials to support home‐based inclusive learning centers.
Genovesi et al. (2024)	To map awareness‐raising and capacity building materials.	Identified a wealth of materials (comics, blogs, videos), but noted a gap in manuals for Eastern European LMICs and resources for intellectual disabilities.

## 4 Discussion

The results of this systematic review point to a multifaceted and interrelated set of barriers, alongside emerging facilitators, that influence the educational experiences of persons with disabilities in low‐ and middle‐income countries (LMICs). Analysis of the 23 included studies reveals a recurring policy–practice divide, where global frameworks such as the United Nations Convention on the Rights of Persons with Disabilities and Sustainable Development Goal 4 are formally endorsed but inadequately enacted in practice. This gap is largely attributable to systemic inertia and chronic underinvestment, both of which constrain the effective translation of policy commitments into accessible and genuinely inclusive learning environments.

### 4.1 Systemic and Environmental Exclusion

A recurring insight across the literature is that mere school enrollment should not be equated with genuine inclusion. This observation aligns with earlier systematic reviews; for example, Bani Odeh and Lach identified a similar gap between enrollment and meaningful participation across diverse global contexts [9], while Egan et al. reported comparable patterns within African LMIC settings [25]. Building on this evidence, the present review offers a more nuanced perspective by illustrating how intersectional factors particularly gender and geographic location shape which students are most likely to remain excluded despite being enrolled.

Although many learners with disabilities are physically present in classrooms, a range of structural barriers continues to limit their active participation. In countries such as India, Ethiopia, and Ghana, students routinely face environmental obstacles, including steep staircases and unsafe access routes. These challenges extend beyond inconvenience, posing significant threats to students' safety and dignity. In some cases, they contribute directly to irregular attendance or complete withdrawal from school, especially for those with mobility impairments who require assistance to navigate the school environment [5, 11, 12].

At the systemic level, limited availability of trained special needs educators, coupled with the absence of essential resources such as Braille materials and sign language interpreters, further constrains effective learning. As a result, many students rely on informal support from peers, which is often inconsistent and insufficient for sustained academic development. In certain contexts, including Azerbaijan [1, 9], these issues are further reinforced by the continued dominance of a medical model of disability, which prioritizes segregated educational settings rather than inclusive mainstream approaches [17].

### 4.2 The Psychosocial and Attitudinal Landscape

Teacher attitudes are far more than just individual biases; they act as fundamental structural barriers to student success. When educators approach disability through a “deficit lens” whether due to ingrained cultural myths or a lack of professional training they often withdraw instructional effort. This creates a self‐fulfilling prophecy, students are denied the necessary support, their performance inevitably dips, and these poor outcomes are then used to justify the original low expectations [1, 19].

Furthermore, research indicates that in many Low‐ and Middle‐Income Countries (LMICs), disability is often interpreted through a tragic or spiritual framework, frequently being dismissed as a divine punishment or a curse [6]. These perceptions fuel peer‐to‐peer bullying and social isolation, leading to what scholar's call “internalized exclusion.” In these cases, even if a student is physically in the classroom, they remain effectively sidelined from both social life and the curriculum [19].

This climate of exclusion extends to the home as well. Many parents remain deeply skeptical of inclusive classrooms, often fearing rightly so that untrained staff will neglect their children. Consequently, many caregivers continue to opt for segregated special schools, favoring the perceived safety of these environments despite their well‐documented shortcomings [28].

### 4.3 Intersectional Modulation of Exclusion

Disability is rarely an isolated factor; rather, it interacts with gender, socioeconomic status, and geography to create unique and often intensified layers of disadvantage. In various African and Asian contexts, for instance, gendered cultural norms often dictate that girls with disabilities are relegated to domestic chores, whereas boys with similar impairments are prioritized for formal education [4, 14].

The COVID‐19 pandemic served as a catalyst that both revealed and worsened these systemic inequities. Students from impoverished backgrounds and specifically girls with disabilities in South Asia and sub‐Saharan Africa faced the most significant hurdles during the transition to remote learning. This was driven not only by a fundamental lack of digital infrastructure and connectivity but also by an increase in domestic responsibilities during periods of school closure [14]. Furthermore, a significant disparity remains between urban and rural service delivery. Children living in remote regions often face delays in disability identification, largely because specialized diagnostic and support services are heavily concentrated in metropolitan hubs. This “detection gap” ensures that the children who are most geographically isolated are frequently the ones with the least access to tailored educational support [2, 16, 18].

### 4.4 Facilitators and the Path Forward

While the obstacles to inclusion are substantial, the existing literature highlights several effective facilitators. Research suggests that the most successful initiatives employ a “twin‐track” strategy: they pair individualized interventions such as targeted literacy and numeracy support with systemic reforms like flexible pedagogical practices and the creation of physically accessible learning environments [8].

Furthermore, assistive technologies have proven to be powerful tools for increasing classroom participation. From high‐tech solutions like augmentative and alternative communication (AAC) devices and interactive apps to essential low‐tech tools like Braille and sign language, these resources significantly improve learning outcomes when they are properly supported and available [8, 16]. Beyond the technical, community led facilitators play a vital role. For example, indigenous values centered on communal responsibility, such as the Ubuntu philosophy found in various Sub‐Saharan African contexts, are frequently credited with fostering a genuine sense of social belonging for students with disabilities [6, 7].

Ultimately, this review suggests that educational exclusion in LMIC contexts cannot be reduced to a single cause [13]. The barriers are simultaneously structural, attitudinal, and intersectional, requiring a multi‐layered response. The evidence clearly indicates that simply adopting a policy or enrolling a student does not guarantee inclusion. Instead, the data points toward the need for intentional, sustained investment in teacher capacity, infrastructure, and community engagement tailored to the specific country, disability type, and the intersecting identities of the students [13]. Notably, a significant methodological gap remains: the voices of the students themselves are largely missing from the current research, a limitation that narrows our interpretive reach and is discussed further in Section 6.

## 5 Practical Implications and Future Research Agenda

Translating high level policy commitments into tangible classroom realities in LMICs requires coordinated action across several sectors. The following recommendations target the specific stakeholders responsible for closing this implementation gap.

*Strategic Recommendations for Stakeholders*

Governments: National authorities should prioritize the development of centralized data repositories. These systems are essential for tracking enrollment, retention, and completion rates specifically for students with disabilities, with data further disaggregated by gender, impairment type, and geography [10, 18] Furthermore, fiscal priority must shift; funds currently consumed by debt servicing should be redirected toward inclusive infrastructure, specialized learning aids, and teacher professional development [7].

Educators: There must be a conceptual shift from “integration” where the child is expected to fit into an inflexible system to “genuine inclusion,” which demands that the system itself be restructured. To support this, mandatory pre‐service and in service training should focus on Universal Design for Learning (UDL) alongside technical skills in Braille, national sign languages, and augmentative and alternative communication (AAC) strategies [7, 19, 27].

Communities: Local programs are needed to dismantle cultural or spiritual misconceptions about disability, replacing them with a rights‐based perspective [9, 28]. Leveraging indigenous values, such as the Ubuntu philosophy, can help cultivate a sense of shared communal responsibility for every child's education [6, 7].

Researchers: The academic community should move toward participatory methodologies. By centering the lived experiences and voices of students with disabilities, researchers can ensure that interventions address actual needs rather than relying on top‐down expert assumptions [9, 16]. This recommendation is consistent with emerging work that has begun to directly capture the perspectives of children and youth with disabilities on their own inclusion for example, syntheses of students' attitudes toward disability [29] and studies examining children's own social, emotional, and academic perceptions of inclusive settings [30] which future LMIC‐focused reviews should draw upon and extend.

### 5.1 A Future Research Agenda

The current body of evidence is disproportionately focused on individual level interventions. To progress, the field must embrace systemic and longitudinal frameworks. We urgently need long term studies that track how inclusive education impacts school completion, graduation, and eventual employment for students with diverse needs [8, 10, 13]. Rather than focusing solely on “fixing” the student, future research should evaluate the efficacy of policy reforms, infrastructural upgrades, and shifts in institutional culture [8]. This is consistent with a recent systematic review of inclusive education interventions in low‐ and lower‐middle‐income countries, which similarly found that rigorous evaluations of teacher training, resource provision, and whole‐school reform remain far scarcer than descriptive accounts of barriers, underscoring the need for more intervention‐focused research in this field [31]. Moreover, data collection must become more granular, accounting for gender, socioeconomic status, ethnicity, and location [8, 14]. There is also a clear need for more evidence from underrepresented regions, particularly Latin America, the Caribbean, and areas affected by conflict or humanitarian crises [8, 16]. Finally, the specific role of assistive technologies ranging from specialized hardware to speech‐to‐text software requires dedicated investigation across different types of impairments [8].

### 5.2 Bridging the Policy‐Practice Gap

Moving from the macro‐level ratification of rights to micro level classroom execution requires accountability mechanisms that are currently missing in most LMIC contexts [2, 10]. Funding should be explicitly earmarked for rural and underserved regions to correct geographic imbalances [10]. Schools should also adopt UDL principles from the outset, designing curricula and physical spaces that are inherently accessible rather than retrofitted as an afterthought [3, 5]. To ensure compliance with national disability laws, decentralized education committees and regular school audits should be established to monitor “inclusion readiness” [10, 12]. Ultimately, sustainable inclusion depends on interdisciplinary partnerships linking health, education, and social services to ensure that medical rehabilitation and educational support work in tandem [2, 8, 16].

## 6 Limitations of the Study

The findings of this review should be interpreted alongside several important constraints within the existing evidence base. Perhaps the most significant limitation is the relative scarcity of firsthand perspectives within our specific sample: most of the 23 included studies rely on proxy data provided by parents, teachers, or institutional representatives rather than the direct lived experiences of people with disabilities themselves. This should not be read as a gap in the wider field, since a small but growing body of research has begun to center the voices of students with disabilities directly [29, 30]; rather, it reflects a limitation of the present search strategy and inclusion criteria, and future updates of this review should deliberately broaden search terms to capture this literature. Furthermore, many of the primary studies are characterized by methodological weaknesses, including small sample sizes and a lack of longitudinal data, which naturally limits how broadly these results can be generalized. There are also notable geographic and thematic gaps, research from humanitarian and conflict affected regions is scarce, and certain conditions such as autism and profound intellectual disabilities remain significantly under researched compared to sensory or physical impairments. Additionally, although this review is registered with PROSPERO (CRD420261360472), registration was completed retrospectively after protocol development rather than prospectively; while no amendments were made to the protocol following registration, this sequence is acknowledged as a limitation in the interest of transparent reporting.

Our search methodology also presented certain challenges to the study's comprehensiveness. We relied primarily on Google Scholar, supplemented by citation tracking and three additional databases. However, the search strategy did not utilize controlled vocabulary indexing (such as MeSH or EMTREE terms) or a formally validated multi‐database protocol. This approach limits the reproducibility of the study and suggests that some relevant literature particularly specialized clinical or nursing studies indexed in MEDLINE or CINAHL may have been overlooked.

Finally, the screening and data extraction processes were conducted primarily by a single reviewer. Although we made every effort to apply the eligibility criteria consistently, the absence of an independent second screener introduces a risk of selection bias that is difficult to quantify. Future versions of this review would benefit from a dual screening process and the reporting of a Cohen's kappa value at both the initial title/abstract stage and the full text review stage to ensure greater methodological intervention.

## 7 Conclusion

This review demonstrates that educational exclusion of persons with disabilities in Low‐ and Middle‐Income Countries results from multiple intersecting systemic challenges rather than from individual impairments alone. International frameworks including the UNCRPD and SDG 4 are widely ratified but inconsistently implemented, a gap driven by chronic underfunding, inadequate teacher preparation, and inaccessible school environments. Negative attitudes, stigma, and peer bullying compound structural barriers by reducing students' confidence and sense of belonging. At the same time, the evidence points to meaningful facilitators, including supportive teaching approaches, assistive technologies, community engagement, and the revitalization of indigenous values of communal responsibility. This review further confirms that gender, poverty, and rural location act as amplifiers of exclusion, disproportionately affecting girls and children from low‐income households with limited access to support services. Addressing these intersecting determinants requires coordinated action across policy, pedagogy, and community systems.

## Author Contributions

The contributions of the authors to this systematic review are outlined below according to the CRediT taxonomy: **Yusufu Simra:** conceptualization, methodology, formal analysis, project administration. **Hussein Mwanjali:** investigation, writing – original draft, writing – review and editing. **Shani Omari:** software, data curation, resources.

## Ethics Statement

This study is a secondary systematic review of previously published literature and did not involve human participants, primary data collection, or animal subjects. As such, formal ethical approval was not required. All included studies were appraised for their own reported ethical compliance during the quality appraisal (MMAT) process.

## Conflicts of Interest

The authors declare no conflicts of interest.

## Transparency Statement

The corresponding author, Yusufu Simra, affirms that this manuscript is an honest, accurate, and transparent account of the study being reported; that no important aspects of the study have been omitted; and that any discrepancies from the study as planned (and, if relevant, registered) have been explained.

## Data Availability

The data supporting the findings of this systematic review are derived from publicly available secondary sources (published literature) cited within the manuscript. The extracted data sheets, quality appraisal results, and the full list of excluded studies generated during the review process are available from the corresponding author (Yusufu Simra) upon reasonable request.

## 1

### 1.1

Quality Appraisal Summary—MMAT 2018

See Table A1.

**Table A1:** presents the quality appraisal ratings for all 23 studies included in this systematic review (references [1, 2, 3, 4, 5, 6, 7, 8, 9, 10, 11, 12, 13, 14, 15, 16, 17, 18, 19, 25, 26, 27, 28]), the same 23 studies described in the Methods and Results sections and summarised in Table 1, assessed using the Mixed Methods Appraisal Tool (MMAT), version 2018. Other works cited elsewhere in this manuscript (e.g., references [20, 21, 22, 23, 24, 29, 30, 31, 32]) are background, theoretical, or comparative sources that informed the Introduction and Discussion but were not identified through the systematic search and screening process described in Section 2; these background references were therefore not part of the included evidence base and were not subject to MMAT quality appraisal. Each study was scored on five criteria appropriate to its design type. Scores are reported as the number of criteria met out of five. Studies scoring 1–2 are rated low quality, 3–4 moderate quality, and 5 high quality. Studies marked with (?) on individual criteria indicate insufficient methodological reporting to confirm criterion met; these were scored as not met.

Ref	Author(s) & year	MMAT design type	Q1	Q2	Q3	Q4	Q5	Score	Rating
[1]	Makwelo et al. (2025)	Quant. descriptive	Y	?	Y	?	Y	3/5	Moderate
[2]	Abebe et al. (2023)	Qualitative	Y	Y	Y	Y	?	4/5	Moderate
[3]	Vergunst & McKenzie (2021)	Qualitative	Y	?	Y	?	?	2/5	Low
[4]	Hussein (2025)	Quant. descriptive	Y	Y	Y	?	Y	4/5	Moderate
[5]	Cosmas (2025)	Qualitative	Y	?	?	?	?	1/5	Low
[6]	Tafirenyika et al. (2023)	Qualitative	Y	Y	Y	Y	Y	5/5	High
[7]	Zickafoose et al. (2024)	Systematic review	Y	Y	?	Y	Y	4/5	Moderate
[8]	Hunt et al. (2025)	Systematic review	Y	Y	Y	Y	Y	5/5	High
[9]	Bani Odeh & Lach (2024)	Systematic review	Y	Y	Y	Y	Y	5/5	High
[10]	Lawal et al. (2025)	Quant. descriptive	Y	?	Y	?	Y	3/5	Moderate
[11]	Gull et al. (2025)	Systematic review	Y	Y	Y	Y	Y	5/5	High
[12]	Rahman & Mehnaz (2024)	Mixed methods	Y	?	?	?	?	1/5	Low
[13]	Miles et al (2018)	Qualitative	Y	Y	Y	Y	?	4/5	Moderate
[14]	Jones et al. (2021)	Mixed methods	Y	Y	Y	Y	Y	5/5	High
[15]	Huus et al. (2021)	Systematic review	Y	Y	Y	Y	Y	5/5	High
[16]	Genovesi et al. (2024)	Systematic review	Y	Y	Y	?	Y	4/5	Moderate
[17]	Abdullayeva (2025)	Qualitative	Y	Y	Y	Y	?	4/5	Moderate
[18]	Vargas et al. (2025)	Quant. descriptive	Y	?	Y	?	Y	3/5	Moderate
[19]	Mbatt & Philip (2024)	Quant. descriptive	Y	Y	Y	?	Y	4/5	Moderate
[25]	Egan et al. (2022)	Systematic review	Y	Y	Y	Y	Y	5/5	High
[26]	Temba et al. (2026)	Qualitative	Y	Y	Y	Y	?	4/5	Moderate
[27]	Kasamba (2018)	Qualitative	Y	?	?	?	?	1/5	Low
[28]	Masuku & Marumolo (2024)	Mixed methods	Y	Y	Y	?	Y	4/5	Moderate

*Note:* Y = criterion met? = insufficient information to confirm criterion met (scored as not met). Q1–Q5 refer to the five MMAT criteria for each respective design type. Quant. = quantitative.
